# Supplementary material for: Predation and fragmentation portrayed in the statistical structure of prey time series
Source: BMC Ecol. 2009 May 6;9:10. doi: 10.1186/1472-6785-9-10 (PMC2689204; doi:10.1186/1472-6785-9-10)
Supplement: Additional file 2 — Voles and related classes ODDox Documentation. ODDox documentation of the agent-based model (ALMaSS) applied by Hendrichsen et al. The documentation is started by activating main.html. [file 1472-6785-9-10-S2.zip › Vole_ODDox/functions_func_0x67.html]

ALMaSS ODDox: Class Members - Functions

- Main Page
- Related Pages
- Classes
- Files

- Alphabetical List
- Class List
- Class Hierarchy
- Class Members

- All
- Functions
- Variables

- a
- b
- c
- d
- e
- f
- g
- h
- i
- k
- l
- m
- n
- o
- p
- r
- s
- t
- u
- v
- w
- ~

### - g -

- GeneticMaterial()
  : GeneticMaterial- GeneticMaterial1616()
    : GeneticMaterial1616- GeneticsResultsOutput()
      : Vole\_Population\_Manager
      , Population\_Manager- GetAllele()
        : GeneticMaterial
        , GeneticMaterial1616- GetDirectFlag()
          : GeneticMaterial
          , GeneticMaterial1616
          , Vole\_Base- GetFarmNumber()
            : Farm- GetFertile()
              : Vole\_Male- GetFirstCrop()
                : CropRotation- GetFirstCropIndex()
                  : Farm- GetFirstDate()
                    : Crop
                    , Farm- GetGeneticFlag()
                      : Vole\_Base
                      , GeneticMaterial
                      , GeneticMaterial1616- GetIntensity()
                        : Farm- GetLocation()
                          : Vole\_Female
                          , Vole\_Base
                          , Vole\_Male- GetNextCrop()
                            : CropRotation- GetNextCropIndex()
                              : Farm- GetNextCropStartDate()
                                : Farm- GetType()
                                  : Farm- Glyphosate()
                                    : Farm- GrowthRegulator()
                                      : Farm

---

Generated on Thu Jan 22 14:13:45 2009 for ALMaSS ODDox by 
 1.5.6
